# Circulating Tumour DNA as Biomarker for Colorectal Liver Metastases: A Systematic Review and Meta-Analysis

**DOI:** 10.3390/cells12212520

**Published:** 2023-10-25

**Authors:** Lissa Wullaert, Jan M. van Rees, John W. M. Martens, Henk M. W. Verheul, Dirk J. Grünhagen, Saskia M. Wilting, Cornelis Verhoef

**Affiliations:** 1 Department of Surgical Oncology and Gastrointestinal Surgery, Erasmus MC Cancer Institute, 3015 GD Rotterdam, The Netherlands; l.wullaert@erasmusmc.nl (L.W.);; 2 Department of Medical Oncology, Erasmus MC Cancer Institute, 3015 GD Rotterdam, The Netherlands

**Keywords:** ctDNA (circulating tumour DNA), cfDNA (circulating free DNA), liquid biopsy, minimal residual disease (MRD), colorectal liver metastases (CRLM)

## Abstract

Circulating tumour DNA (ctDNA) is a potential biomarker that could contribute to more judicious patient selection for personalised treatment. This review and meta-analysis gives an overview of the current knowledge in the literature investigating the value of ctDNA in patients with colorectal liver metastases (CRLM). A systematic search was conducted in electronic databases for studies published prior to the 26th of May 2023. Studies investigating the association between ctDNA and oncological outcomes in patients undergoing curative-intent local therapy for CRLM were included. Meta-analyses were performed to pool hazard ratios (HR) for the recurrence-free survival (RFS) and overall survival (OS). A total of eleven studies were included and nine were eligible for meta-analyses. Patients with detectable ctDNA after surgery experienced a significantly higher chance of recurrence (HR 3.12, 95% CI 2.27–4.28, *p* < 0.000010) and shorter OS (HR 5.04, 95% CI 2.53–10.04, *p* < 0.00001) compared to patients without detectable ctDNA. A similar association for recurrence was found in patients with detectable ctDNA after the completion of adjuvant therapy (HR 6.39, 95% CI 2.13–19.17, *p* < 0.0009). The meta-analyses revealed no association between detectable ctDNA before surgery and the RFS and OS. These meta-analyses demonstrate the strong association between detectable ctDNA after treatment and oncological outcomes in CRLM patients.

## 1. Introduction

Treatment strategies in colorectal cancer care have been evolving greatly in recent years. Still, about 30–40% of patients will eventually develop metastatic disease, with the liver being the most common site [1,2]. Approximately 25% of patients with colorectal liver metastases (CRLM) undergo treatment with curative intent through local therapies, with around 40% of these patients facing recurrence of their disease within one year after surgery (early recurrence) [3,4,5,6,7]. To improve the outcomes in patients with CRLM, novel biomarkers are needed to contribute to more judicious patient selection for personalised treatment.

One of these new biomarkers with high potential is circulating tumour DNA (ctDNA), fragmented tumour-derived DNA, released in the blood stream through cancer cell apoptosis, necrosis and phagocytosis. Other mechanisms leading to ctDNA release are active secretion in extracellular vesicles and mitochondrial DNA egestion [8,9]. It is hypothesised that ctDNA reflects the tumour burden and may therefore be a novel instrument for measuring minimal residual disease (MRD) after treatment with curative intent [10]. Compared to carcinoembryonic antigen (CEA), ctDNA has the advantage of a short half-life, providing real-time molecular information to monitor the treatment response and detect potential relapse, and may therefore give valuable insight into a patients’ prognosis. Furthermore, CEA is known to have a moderate sensitivity in recurrence detection and has the disadvantage of having relatively high rates of false positivity [11,12].

Several techniques can be used to detect MRD with ctDNA in peripheral blood samples. One common approach to detect ctDNA is with the use of a predefined panel of mutations commonly found in (colorectal) cancer. Herein, the mutation status of the tumour is not warranted, and is therefore usually referred to as the tumour-agnostic approach. Conversely, the tumour-informed approach entails prior analysis of individual tumour tissue to identify specific alterations. This technique allows for more focused mutation tracking in the plasma samples, and has the advantage of having a higher specificity compared to the tumour-agnostic approach. Identifying the mutation profile can either be achieved by sequencing the whole genome of a tumour, but may also be adopted from the use of a set of the most common colorectal cancer mutations.

The aim of this systematic review and meta-analysis is to give an overview of the current knowledge in the literature on the prognostic value of ctDNA in patients with CRLM. Even though the role of ctDNA has been studied widely in primary colorectal cancer, knowledge on the long-term oncological survival outcomes of patients with or without ctDNA in the metastatic setting is still preliminary. To expand the knowledge on this topic, a meta-analysis pooling long-term oncological survival outcomes of patients with CRLM with and without detectable ctDNA preoperatively, postoperatively, and at the end of adjuvant chemotherapy was performed.

## 2. Materials and Methods

In this study, the PRISMA (Preferred Reporting Items for Systematic Reviews and Meta-analysis, www.prisma-statement.org) guidelines were followed. The meta-analysis was registered in the international prospective register of systematic reviews (PROSPERO CRD42023453375). A search was conducted in Pubmed/Medline, Embase, Web of Science, the Cochrane database, and Google Scholar, for studies published prior to the 26th of May 2023. The search terms are provided in Supplementary Materials File S1. Two authors screened eligible studies independent of each other (L.W. and J.R.), and joint assessment was performed in case of disagreement. Observational cohort studies and randomised controlled trials investigating the prognostic value of ctDNA in patients with liver-only metastases undergoing curative-intent resection were included. Trials investigating circulating tumour cells (CTCs) and disseminated tumour cells (DTCs) extracted from bone marrow were excluded. Case reports, conference abstracts, non-original studies and studies written in any language other than English were excluded. Reference lists were screened to identify additional eligible articles.

### 2.1. Data Extraction and Quality Assessment

Data on study design, population characteristics, detection methods and oncological outcomes were collected. Quality assessment was conducted by two reviewers (L.W. and J.R.) and a third reviewer (S.W.) was involved when disagreement occurred. Studies were categorised by low, moderate, and high risk of bias, using the validated Quality In Prognosis Studies (QUIPS) tool [13,14] and the outcomes thereof visualised using the risk-of-bias VISualization (robvis) tool [15].

### 2.2. Quantitative Assessment

Meta-analyses and figures were conducted and designed through Review Manager (RevMan) version 5.4.1, The Cochrane Collaboration, 2020 [16]. Hazard ratios (HRs) and 95% confidence intervals were extracted from the included articles. Studies were excluded from meta-analyses when they failed to report HRs, after scrutinising available supplementary data. The generic inverse-variance method using a random-effects model was utilised to perform the meta-analyses on recurrence-free survival (RFS) and overall survival (OS). Results were presented as hazard ratios (HRs) with corresponding *p*-values or 95% confidence intervals (CI). Heterogeneity was assessed using the I^2^ statistic. To evaluate robustness of results, sensitivity analyses were performed by excluding studies scored as high risk of bias. Funnel plot analysis was performed to detect publication bias.

## 3. Results

### 3.1. Literature Search

**Figure 1:**
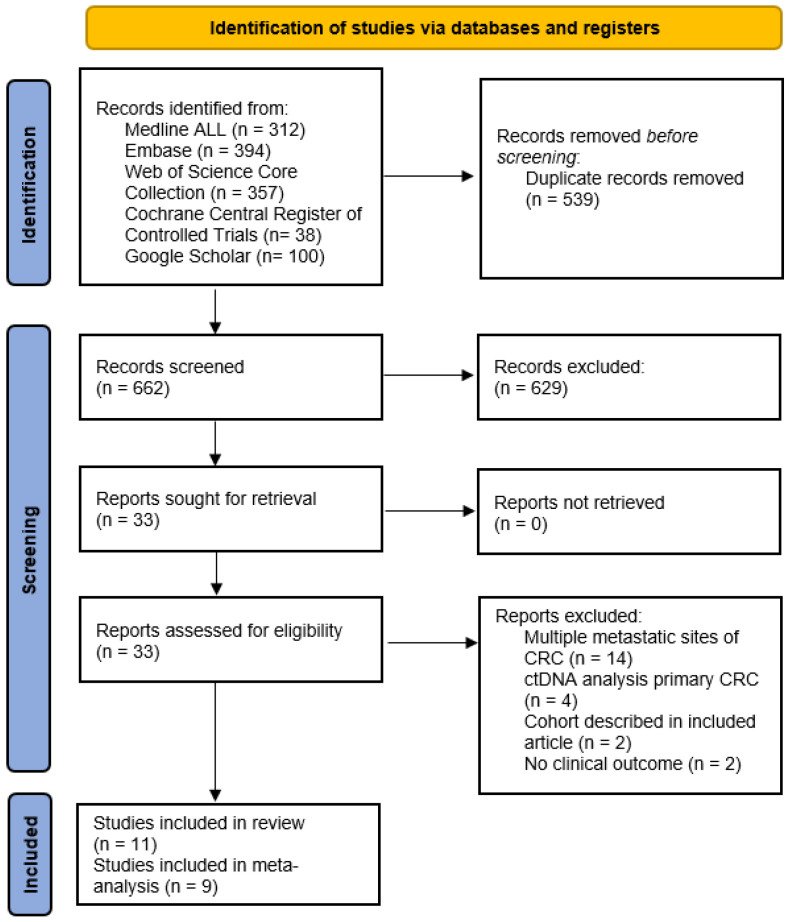
PRISMA flowchart.

The literature search resulted in a total of 662 relevant articles after removal of 539 duplicates. The screening and selection process is illustrated in the PRISMA flowchart in Figure 1. Six hundred and twenty-nine records were excluded after title and abstract screening, resulting in thirty-three reports for full text screening. Ultimately, a total of eleven studies were eligible for inclusion in this systematic review and nine could be used to conduct meta-analyses. The exclusions were due to multiple metastatic sites of colorectal cancer patients (*n* = 14), ctDNA analysis in the primary tumour setting (*n* = 4), and no clinical outcome (*n* = 2). A total of two studies were excluded because they belonged to the same authors as the study of Ogaard et al. [17]. These studies included patients with CRLM in the Aarhus University Hospital with the same set of in- and exclusion criteria and within the same time frame. As Ogaard et al. is the most recent publication and has the largest group of patients, samples and time frame, this study was included in the meta-analyses. Reinert et al. and Scholer et al. are addressed in the results section [18,19].

### 3.2. Overall Study Characteristics

Eleven studies assessing the prognostic value of ctDNA in patients with CRLM were included, of which six were prospective single centre studies [17,20,21,22,23,24], three were prospective multicentre studies [25,26,27], of which two were part of a larger randomised controlled trial [25,26], and lastly, two analysed patients in a retrospective manner [28,29]. The combined sample size resulted in a total of 745 patients undergoing curative-intent treatment for CRLM, ranging from 20 to 153 patients per study. The overall study characteristics and risk of bias are shown in Table 1.

After the quality assessment, a total of three studies (27%) were considered to have a low risk of bias [20,24,27]. The majority were considered to have a moderate risk of bias (45%) [17,23,25,26,28] and three (27%) scored with a high risk of bias [21,22,29]. A high risk of bias was mostly based on a lower quality in attrition and confoundment, as depicted in the figures in Supplementary Tables S1 and S2. A variety of quantification methods and assays were utilised, of which next generation sequencing (NGS) and digital polymerase chain reaction (dPCR) were the most common techniques. An overview of the methods for ctDNA analysis is shown in Table 1 and more detailed information (tube type, pre-processing, plasma and ctDNA isolation) is available in Supplementary Table S3. Additionally, a visual representation of the sample timing, ctDNA detection rates, and perioperative treatment is given in Supplementary Figure S1.

### 3.3. Studies Using a Tumour-Informed ctDNA Approach

A total of five studies performed a tumour-informed approach [23,25,26,27,29], of which one used a personalised assay based on the mutations of the primary tumour [27] and two focused on the blood samples collected in the preoperative setting, which is discussed in a separate section [21,26].

Tie et al. investigated ctDNA in a cohort receiving upfront resection with adjuvant chemotherapy and a cohort receiving perioperative chemotherapy [27]. A baseline sample was collected for both cohorts before administration of any treatment, showing detectable ctDNA in 46 of 54 (85%) patients at baseline. A non-significant trend for a shorter RFS was found for patients who had detectable ctDNA at baseline (HR 4.7, 95% CI 0.63–35.10) and no difference was seen in OS (HR 1.4, 95% CI 0.13–6.18). Cohort 2 received neoadjuvant treatment for which serial sampling was conducted, whereas cohort 1 underwent liver resection without any neoadjuvant treatment. A postoperative sample was taken for both cohorts, in which detectable ctDNA was found in 12 out of 49 (24%) patients after surgery using a tumour-informed approach. Personalised assays derived from each patient’s tumour tissue, using a total of 15 genes commonly mutated in colorectal cancer, were designed to identify ctDNA in the plasma. Patients with postoperative detectable ctDNA, with and without neoadjuvant chemotherapy, or detectable ctDNA at the end of all treatment, after completion of adjuvant chemotherapy treatment, had a significantly shorter RFS and OS compared to those without detectable ctDNA. The reported RFS rates at five years were 17% and 69% for patients with- and without detectable ctDNA after surgery, respectively (HR 6.3, 95% CI 2.6–15.2), and 0% and 75.6% for patients with and without detectable ctDNA at the end of treatment, respectively (HR 14.9, 95% CI 4.94–44.70). After the correction for confounding factors, the postoperative ctDNA status remained an independent predictor for RFS (HR 3.13; 95% CI 1.00–9.82). The five-year OS estimates were 31.7% and 77.7% for, respectively, the group with postoperative detectable ctDNA and the undetectable ctDNA groups (HR 4.2, 95% CI 1.50–11.80).

### 3.4. Studies Using Single Gene ctDNA Status

The CAIRO 5 study by Bolhuis et al. is a randomised phase 3 trial of the Dutch Colorectal Cancer Group (DCCG), investigating the most effective first-line systemic regimens of chemotherapy plus targeted therapy in 297 patients with initially unresectable RAS-mutant CRLM [25]. Out of the 59 RAS-positive patients that eventually received liver resection, baseline, pre-, and postoperative samples were available for a total of 23 patients. Ten (44%) patients received doublet chemotherapy (FOLFOX or FOLFIRI) plus bevacizumab, and 13 (57%) patients received triplet chemotherapy (FOLFOXIRI) plus bevacizumab. The ctDNA analysis was performed using droplet digital PCR (ddPCR) for KRAS and NRAS mutations extracted from plasma. The baseline and preoperative samples were not analysed in terms of the association with disease recurrence. The analyses of the postoperative blood samples showed that six (26%) patients had detectable KRAS or NRAS mutations in peripheral blood samples after neoadjuvant chemotherapy and surgery, which was considered ctDNA-positive. There was an association between postoperative detectable ctDNA status and RFS (HR 3.3, 95% CI 1.1–9.6), which remained strong in the sensitivity analysis adjusting for a set of variables in a multivariable model (HR 4.1, 95% CI 1.19–14.47). Additionally, the postoperative ctDNA status was strongly correlated with the pathologic response to neoadjuvant chemotherapy (*p* < 0.001). All patients (*n* = 15) with undetectable ctDNA had a partial or major pathologic response in the resected tissue from the CRLM, compared to only one patient with detectable ctDNA (*p* < 0.001).

Polivka et al. investigated KRAS-mutated ctDNA in a retrospective cohort of 71 CRLM patients receiving resection and adjuvant treatment [29]. This study focused mainly on the concordance between the ctDNA sample mutations and tissue-based KRAS mutation status. The authors also compared the disease-free survival (DFS) and the OS in patients who had detectable KRAS before surgery in plasma using the dPCR detection technique. Preoperative plasma samples were available of 30 patients who had KRAS-positive liver metastases of which 11 (37%) had KRAS-positive ctDNA samples. There was a trend towards a shorter DFS (median 357 vs. 470 days, *p* = 0.074) after liver surgery in patients who had a KRAS-positive vs. -negative preoperative sample, but no difference in the OS (median 1269 versus 1390 days, *p* = 0.234). A high level of preoperative plasma KRAS fractional abundance (cut-off 3.33%) was an independent negative prognostic factor for the OS after liver surgery, also in the multivariate analysis (HR 44.87, 95% CI 1.59–1266.48).

Narayan et al. included 59 patients in a prospective manner, of whom 64% received preoperative systemic treatment and all underwent adjuvant treatment [23]. They collected plasma samples during surgery before liver manipulation (peripheral sample), and prior to hepatic resection, samples were drawn from the hepatic vein and portal vein. Additionally, a postoperative sample was collected within three to four weeks after surgery. The samples with detectable ctDNA from the hepatic and portal veins were not significantly associated with long-term outcomes. The authors looked more closely at the two most commonly mutated genes: TP53 and APC. In the peripheral blood sample, no significant associations were found for TP53 (*p* = 0.66), APC (*p* = 0.06), or any ctDNA (*p* = 0.58) with DFS. Postoperative samples were, however, not reported in terms of the long-term outcomes.

### 3.5. Studies Using a Tumour-Agnostic ctDNA Approach

The most recently published retrospective cohort study was by Nishioka et al. and detected postoperative ctDNA in 30% of 105 patients who underwent hepatectomy [28]. The ctDNA was analysed using a next-generation sequencing panel of 70 cancer-related genes, and the ctDNA positivity was defined as the presence of one or more gene mutations in plasma. Most patients had synchronous CRLM (74%) and multiple CRLM (64%) and underwent preoperative chemotherapy (86%). Adjuvant treatment was considered individually, but the proportion of patients who underwent postoperative chemotherapy was not reported. Multivariate analyses concluded a significantly shorter RFS for patients with detectable postoperative ctDNA (HR 2.04, 95% CI 1.18–3.52). The authors also investigated early recurrence, which was defined as having recurrence within 1 year after hepatectomy. A total of 66 patients (63%) experienced early recurrence. Of 32 patients with detectable postoperative ctDNA, 30 (94%) experienced early recurrence, compared with 36 patients (49%) without detectable postoperative ctDNA (*p* = 0.003). This resulted in a sensitivity of the detectable postoperative ctDNA for early recurrence of 45.5%, and a specificity of 94.9%. The positive predictive value of detectable postoperative ctDNA for early recurrence was 93.8%, and the negative predictive value was 50.7%. The postoperative ctDNA was the only factor independently associated with early recurrence in their multivariate analysis (OR 11.8, 95% CI 2.32–59.8).

Newhook et al. conducted a prospective cohort analysis and found that 34 out of the 48 patients (71%) had detectable ctDNA prior to hepatectomy [20]. Of the 48 patients, 42 (88%) underwent neoadjuvant chemotherapy and twenty-five patients (52%) underwent adjuvant chemotherapy. Three approaches to define the postoperative ctDNA positivity were assessed, namely a tumour-agnostic approach identifying variant allele frequency (VAF) in plasma, a tumour-informed approach, and a variant classifier approach. The latter was used to increase the specificity of a plasma-only agnostic approach, in which ctDNA profiles are used to train the classifier to differentiate between tumour-derived alterations from non-tumour-derived alterations. There was no association between the preoperative ctDNA detection and the RFS (HR 1.23, 95% CI 0.59–2.6) or OS (HR 2.16, 95% CI 0.71–6.5). A total of 18 patients (38%) had detectable ctDNA postoperatively. Postoperative ctDNA detection using the agnostic variant classifier technique had the highest sensitivity (52.9%) and specificity (100%) for recurrence. Postoperative ctDNA status was significantly associated with both RFS (median 7.5 months for detectable ctDNA vs. 33.0 months for undetectable ctDNA, *p* = 0.0005) and OS (median 42.0 months for detectable ctDNA vs. not reached for undetectable ctDNA, *p* = 0.015). On univariate analysis, postoperative ctDNA detection was associated with a shorter RFS (HR 3.23, 95% CI 1.6–6.6). Perioperative ctDNA dynamics were also investigated in this study. The RFS was significantly shorter for patients with ctDNA detected both before and after surgery, compared to patients with no ctDNA detected before and after surgery (HR 0.34, 95% CI 0.14–0.86), and patients with ctDNA clearance after surgery (HR 0.24, 95% CI 0.10–0.58).

The largest prospective trial in terms of samples was conducted by Ogaard et al., including 96 patients undergoing CRLM resection with a total of 499 collected plasma samples [17]. They detected ctDNA in 97.6% (82/84) of patients with preoperative plasma available using a tumour-agnostic methylation multiplex ddPCR test and reported no association between the preoperative ctDNA level and recurrence (Fisher’s exact; *p* = 0.9999). The authors included 96 patients undergoing CRLM resection, 9 patients received neoadjuvant treatment and 42 patients received additional adjuvant chemotherapy. Postoperative ctDNA was detected in 65% of patients. Patients with ctDNA postoperatively or after the completion of adjuvant chemotherapy experienced a significantly shorter RFS than patients without detected ctDNA (HR 4.5, 95% CI 2.1–9.5 and HR 8.4, 95% CI 3.1–23.1). Methylation-based ctDNA positivity in postoperative peripheral blood samples was also a strong predictor of recurrence assessed solely for patients treated without adjuvant chemotherapy (HR 4.7, 95% CI 1.9–11.8). In the multivariable analysis, the postoperative cDNA status remained significantly associated with the RFS (HR 5.3, 95% CI 2.1–13.0). The authors at the Aarhus University Hospital previously published two other prospective observational studies and conducted analyses of the same patient cohort with the same in- and exclusion criteria and time frame. Therefore, these studies were not included in the meta-analyses. Scholer et al. prospectively enrolled a total of 118 colorectal cancer patients, but also included a validation group of 18 colorectal cancer patients with metachronous (fifteen patients) or synchronous (three patients) liver metastases [19]. Of these patients, eleven patients received adjuvant therapy. A single plasma sample was collected at three months after surgery and the KRAS mutation status was investigated on KRAS. ctDNA-positive patients showed a very high risk of relapse (HR 4.9, 95% CI 1.5–15.7). Reinert et al. analysed a cohort of 68 patients with CRLM and investigated the following mutations through ddPCR: APC, BRAF, KRAS, NRAS, PIK3CA, and TP53 [18]. The detection of ctDNA after surgery was associated with a reduced RFS as compared to patients with no detectable ctDNA after surgery (HR 7.6, 95% CI 3.0–19.7). The 2-year RFS was 2.9% (1 of 34 patients) for the patients with detectable ctDNA and 24.2% (8 of 33 patients) for the patients with undetectable ctDNA (HR 4.3, 95% CI 2.2–8.1).

Wang et al. included a total of 84 patients who received preoperative chemotherapy for CRLM, of which 83 patients received adjuvant chemotherapy [24]. ctDNA in plasma was defined as positive when the VAF was ≥0.5% in a ctDNA 451-gene NGS panel. The results show that a 10-fold decrease in the VAF after neoadjuvant therapy predicted a significantly better tumour response (*p* = 0.004). ctDNA detection in baseline (*p* = 0.152) and preoperative (*p* = 0.232) samples was not significantly associated with RFS. However, patients with undetectable postoperative ctDNA had a significantly longer RFS than those with detectable ctDNA (HR 0.37, 95% CI 0.21–0.68). After receiving adjuvant chemotherapy, this trend sustained with a significantly longer RFS compared to those with detectable ctDNA post-adjuvant chemotherapy (HR 0.41, 95% CI 0.19–0.87). Both remained an independent predictor of the RFS after adjusting for known clinicopathological risk factors, e.g., the nodal involvement of the primary tumour and the number of CRLM.

He et al. investigated the prognostic value of ctDNA and cfDNA in a cohort of 20 colorectal cancer patients with CRLM, of which eleven patients received neoadjuvant chemotherapy and 16 patients underwent adjuvant chemotherapy [22]. Samples were collected before and after surgery and were considered positive if one of the genes in the Coloncore panel, which covers all exons of 41 colon cancer-related genes, was mutated. Furthermore, they investigated the possible association between the tumour volume of liver metastases and CEA, cfDNA, and ctDNA. The results show that low presurgical ctDNA levels experienced a longer PFS (*p* < 0.001). Four patients had detectable ctDNA after surgery and no association was found between the detection of ctDNA postoperatively and the progression-free survival (*p* = 0.472).

### 3.6. Studies Investigating Only Preoperative ctDNA Samples

Bidard et al. conducted sub-analyses in the PRODIGE-14 trial, in which patients with CRLM ineligible for curative resection were allocated in chemotherapeutic treatment groups based on the KRAS exon 2 mutational status in their tumour tissue [26]. The ctDNA analysis was performed at baseline before any treatment, after one month of systemic therapy and before surgical resection of patients with a known exon 2 KRAS mutation. The samples with a VAF of >0.1% were classified as ctDNA-positive. Among the 46 patients with a KRAS exon 2 mutated tumour, KRAS-mutated ctDNA was detected at baseline in 42 patients (91%). During neoadjuvant chemotherapy, the KRAS ctDNA levels significantly decreased: at four weeks, a total of 63% of patients with KRAS-mutated tumours had detectable ctDNA, while this dropped to 19% of patients preoperatively (*p* = 0.0001). ctDNA detection at 4 weeks after commencement of neoadjuvant chemotherapy had no prognostic impact (*p* = 0.31). Among the 17 patients referred to resection of CRLM after neoadjuvant therapy, the detection of ctDNA preoperatively (*n* = 4) was significantly associated with a short OS (HR 31, 95% CI 3.2–317).

Kobayashi et al. found that in a multicentre observational cohort of 212 patients, 32 patients (80%) out of 40 with preoperative blood samples available had detectable ctDNA, using a commercially available comprehensive genotyping assay that detects variants in 74 genes (Guardant360^®^) [21]. A total of five patients underwent preoperative chemotherapy and 21 patients received adjuvant treatment. The results show that 20 of 32 patients with detectable ctDNA before CRLM resection developed recurrence, while only one patient without detectable preoperative ctDNA experienced recurrence in the follow-up period (20/32 (63%) and 1/8 (13%), respectively (OR 11.0, 95% CI 1.2–549.6)). A shorter RFS was shown in patients with detectable ctDNA compared to patients without detectable ctDNA among patients with liver-limited metastases (HR 6.8, 95% CI 0.9–51.4).

### 3.7. Meta-Analyses

#### 3.7.1. Recurrence-Free Survival (RFS)

**Figure 2:**
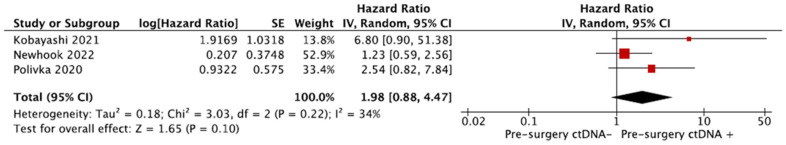
Meta-analysis of the association between recurrence-free survival and the presence of ctDNA preoperatively [20,21,29]. The pooled hazard ratio (black diamond) for ctDNA presence before surgery in CRLM patients is 1.98 (95% CI 1.04–4.36; *p* = 0.10) compared to patients without detectable ctDNA. A hazard ratio of the study result is represented by a red box. The boxes’ size represents the study’s weight in the analysis. The horizontal line acts as the 95% confidence intervals of the study result, with each end of the line representing the boundaries of the confidence interval. (HR hazard ratio, CI confidence interval).

A total of three studies were eligible for the assessment of the RFS of patients with detectable versus without detectable ctDNA preoperatively [20,21,29]. The pooled hazard ratio for ctDNA presence before surgery in CRLM patients was 1.98 (95% CI 1.04–4.36; *p* = 0.10) compared to patients without detectable ctDNA (Figure 2). Tie et al. included two cohorts, one with patients receiving neoadjuvant chemotherapy, in which the preoperative sample was taken before any treatment (T0), and one cohort without any neoadjuvant chemotherapy, in which the first sample was taken before resection (T0) [27]. The preoperative sample of the neoadjuvant therapy cohort was therefore more of a baseline sample than a true preoperative sample (Supplementary Figure S1). As these samples were analysed together, Tie et al. was not included in this meta-analysis. A sensitivity analysis was conducted by removing the studies considered to have a high risk of bias to test for the robustness of results [21,29]. This resulted in no additional findings (HR 1.23, 95% CI 0.59–2.56; *p* = 0.58).

**Figure 3:**
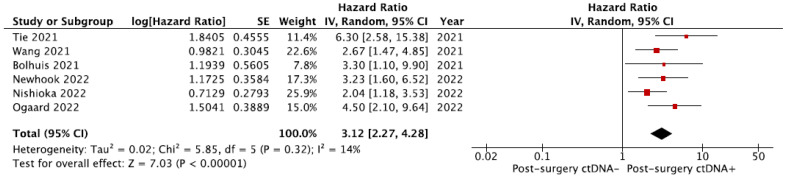
Meta-analysis of the association between recurrence-free survival and the presence of ctDNA after curative-intent surgery [17,20,24,25,27,28]. The pooled hazard ratio for ctDNA presence after surgery in CRLM patients is 3.12 (95% CI 2.27–4.28; *p* < 0.00001) compared to patients without detectable ctDNA.

A total of six studies were eligible for the quantitative assessment of the association between the detectable ctDNA postoperatively and RFS [17,20,24,25,27,28]. The pooled hazard ratio for ctDNA presence after surgery in CRLM patients was 3.12 (95% CI 2.27–4.28; *p* < 0.00001) compared to patients without detectable ctDNA (Figure 3).

**Figure 4:**
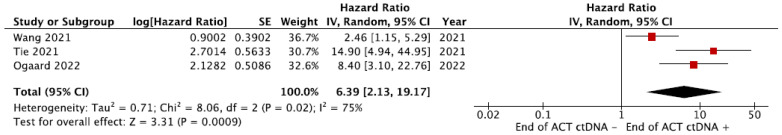
Meta-analysis of the association between recurrence-free survival and the presence of ctDNA after end of adjuvant treatment [17,24,27]. The pooled hazard ratio for ctDNA presence at the end of adjuvant chemotherapy in CRLM patients was 6.39 (95% CI 2.13–19.17; *p* = 0.0009) compared to patients without detectable ctDNA.

Three studies were eligible for the assessment of the RFS of patients with detectable versus without detectable ctDNA at the end of adjuvant therapy [17,24,27]. The pooled hazard ratio for ctDNA presence at the end of adjuvant chemotherapy in CRLM patients was 6.39 (95% CI 2.13–19.17; *p* = 0.0009) compared to patients without detectable ctDNA (Figure 4). There was a high degree of heterogeneity (75%).

#### 3.7.2. Overall Survival (OS)

**Figure 5:**
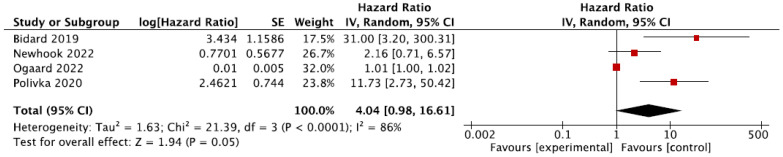
Meta-analysis of the association between overall survival and the presence of ctDNA preoperatively [17,20,26,29]. The pooled hazard ratio for ctDNA presence before surgery in CRLM patients was 4.04 (95% CI 0.98–16.61; *p* = 0.05) compared to patients without detectable ctDNA.

A total of four studies were eligible for the analysis of the preoperative sample in terms of the overall survival [17,20,26,29]. The pooled hazard ratio for ctDNA presence before surgery in CRLM patients was 4.04 (95% CI 0.98–16.61; *p* = 0.05) compared to patients without detectable ctDNA, as shown in Figure 5. There was a high degree of heterogeneity (86%). A sensitivity analysis was conducted by removing the studies considered to have a high risk of bias [29]. This resulted in no additional findings (HR 2.64, 95% CI 0.66–10.51; *p* = 0.17).

**Figure 6:**
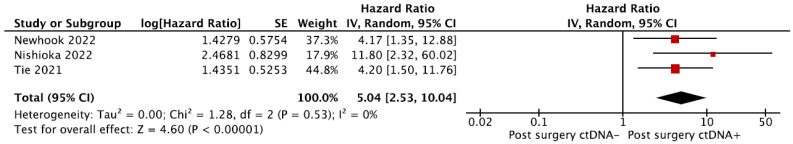
Meta-analysis of the association between overall survival and the presence of ctDNA after curative-intent surgery [20,27,28]. The pooled hazard ratio for ctDNA presence after surgery in CRLM patients is 5.04 (95% CI 2.53–10.04; *p* < 0.00001) compared to patients without detectable ctDNA.

Patients with ctDNA presence after surgery had a shorter overall survival compared to patients without detectable ctDNA (HR 5.04, 95% CI 2.53–10.04; *p* = 0.00001) (Figure 6) [20,27,28].

The risk of publication bias is graphically summarised in and visually estimated through funnel plots (Supplementary Figure S2), in which we found no indication for publication bias in the RFS and OS.

## 4. Discussion

This systematic review aimed to provide a comprehensive overview on the available literature and evidence on the relevance of ctDNA in patients with CRLM. Most studies reported a positive association between the postoperative ctDNA status and the RFS and OS. All the included studies were published after 2017, indicating that ctDNA is a relatively new biomarker in the field of CRLM. Though the role of ctDNA in primary colorectal cancer has been widely studied, our understanding of the role of ctDNA in the metastatic setting remains preliminary. This systematic review and meta-analysis expands the knowledge on this topic in patients with CRLM.

The current study found a hazard ratio for ctDNA presence after surgery in CRLM patients of 3.12 (95% CI 2.27–4.28) for recurrence and of 5.04 (95% CI 2.53–10.04) for overall survival. Faulkner et al. reported similar hazard ratios for the overall survival in the primary colorectal cancer setting, in which more extensive ctDNA research has been conducted [30]. On the other hand, the hazard ratio for recurrence seems to be lower for colorectal cancer patients. For example, a recent meta-analysis conducted by our group regarding rectal cancer patients undergoing neoadjuvant treatment and surgery showed an association between detectable ctDNA after surgery and disease recurrence (HR 15.54, CI 95% 8.23–29.34), marking its importance in the primary setting [31]. Another recently published systematic review showed a progression-free survival with a hazard ratio of 7.93 (95% CI 4.27–14.75) in a subgroup meta-analysis of postoperative ctDNA following surgery for primary colorectal cancer, in favour of a postoperatively negative ctDNA status [30]. In the metastasised setting, this difference was still significant but to a lesser extent (HR 4.58, 95% CI 2.26–9.28). Similarly, Jones et al. explored ctDNA as a prognostic marker in patients with stage IV colorectal cancer, showing a correlation between detectable ctDNA after curative treatment and overall survival (HR 2.2, 95% CI 1.79–2.69), as well as the progression-free survival (HR 3.15, 95% CI 2.10–4.73), in resectable metastatic disease [32].

In contrast with primary colorectal cancer, relatively high recurrence rates are seen in CRLM patients without detectable ctDNA (3-year recurrence rates of approximately 30%, compared to 12% in primary colorectal cancer) [27,33]. This explains the relatively low hazard ratios found in CRLM, and may also indicate that the prognostic value of a ctDNA-negative result is limited in the metastatic setting. Thus, the higher a priori chance of recurrence after resection, also in patients without detectable ctDNA, could diminish the predictive value of postoperative ctDNA in metastatic patients. On the other hand, patients who have detectable ctDNA after surgery will almost always recur, especially in the metastatic setting [20,28]. Therefore, the presence of ctDNA after local treatment of CRLM seems to be a very good predictor for recurrence, whereas the absence of ctDNA may not be as meaningful yet in terms of oncological outcomes. This is well demonstrated in the study of Nishioka et al., which showed that of all patients with early recurrence, defined as recurrence within one year after CRLM resection, 95% had detectable ctDNA after surgery [28].

The included studies investigating the prognostic value of a preoperative sample had divergent results with Kobayashi et al. and Polivka et al. suggesting an association between preoperative ctDNA detection and both the RFS and OS [21,29]. On the other hand, Newhook et al. found no association between prehepatectomy ctDNA detection and survival [20]. The meta-analysis of this current study investigating the association between the RFS and the presence of ctDNA preoperatively seemed to show a significant difference at first, resulting in a hazard ratio of 2.13 (95% CI 1.04–4.36; *p* = 0.04) for ctDNA presence compared to patients without detectable ctDNA. However, after conducting a sensitivity analysis this association did not persist (HR 1.98, 95% CI 1.04–4.36; *p* = 0.10). Additionally, no convincing association was found between the overall survival and ctDNA presence before surgery in CRLM patients (HR 4.04, 95% CI 0.98–16.61; *p* = 0.05) compared to patients without detectable ctDNA. Therefore, of the available evidence, though limited, no definite association could be found between the preoperative ctDNA status and prognosis of patients with CRLM.

The ctDNA detection at baseline (before any treatment) of the studies included in this review was 85–89% [24,27], whereas the ctDNA positivity after neoadjuvant treatment was between 33% and 98% [17,20,21,22,23,24,26,29]. This differed due to the amount of patients who underwent neoadjuvant treatment and the investigated mutations. After CRLM resection, the rate dropped to 20–65% [17,20,22,24,25,27,28]. Although the ranges were mostly similar, patients received heterogeneous treatment strategies and many different ctDNA detection methods were used. A recent study by Wang et al. showed the impact of neoadjuvant chemotherapy on ctDNA in CRLM patients and revealed that early ctDNA dynamic changes can accurately predict the neoadjuvant treatment response and recurrence [34]. This study showed that after one cycle of neoadjuvant treatment, the ctDNA VAF declined remarkably (*p* < 0.0001) and the early changes in the ctDNA VAF, but not in CEA or CA19-9, were an independent indicator of the RFS (HR 4.0, 95% CI 1.22–13.15). As opposed to the results from Tie et al., although showing a median 41-fold decrease in the ctDNA levels during neoadjuvant treatment, the ctDNA clearance during neoadjuvant chemotherapy showed no reduced risk of recurrence following surgery [27]. The limitations of these studies are their relatively small sample sizes.

The advantages of a tumour-agnostic approach are lower costs and a shorter turnaround time, as there is no necessity to collect and analyse tumour tissue. On the other hand, there is a potential risk of missing important genetic alterations that are not commonly found in colorectal cancer patients, which may have important clinical implications for false-negative patients. This may explain the high rates of recurrence in patients without detectable ctDNA after CRLM resection. A more personalised technique is the tumour-informed approach, which could improve the ctDNA detection rate by identifying less commonly mutated genes from the specimen [35]. This technique may eventually have more clinical utility in adjuvant therapy decision-making and the assessment of treatment response. Other novel methods gaining interest in the ctDNA field are DNA methylation and fragmentomics, which are potentially able to accurately detect disease activity in patients’ blood without the need for informed analysis using tumour tissue [17,36,37,38].

A variety of mutation panels are available for the detection of ctDNA in colorectal cancer patients, like the Guardant 360 and Oncomine colon panel, and can be tested directly on tumour tissue or in blood samples [21,39]. These panels consist of different numbers of genes, but are all designed to detect mutations in a set of genes commonly associated with colorectal cancer, such as APC, TP53, KRAS, and BRAF. A relatively new assay, the Guardant Reveal, combines the epigenomic signatures related to aberrant DNA methylation in addition to “standard” detection of common tumour-derived genomic alterations in colorectal cancer patients [40]. The sensitivity of any mutation panel can be affected by factors such as the sample type (blood versus tissue), tumour heterogeneity, low tumour burden, and the presence of cfDNA in the sample, and may vary depending on the specific mutations of interest. Tie et al. showed in their cohort that using a 15-gene panel may well be sufficient to detect at least one clonal mutation in all colorectal tumour tissues [27]. Once the mutations of interest have been found in tissue, a postoperative blood sample using, e.g., dPCR, can be used to detect ctDNA in plasma. This is, however, a small cohort and the accompanied low tumour fraction and limited number of DNA fragments imposes a limit on low disease burden monitoring. Laundau et al. proposed complementing breadth with depth of sequencing by whole genome sequencing, allowing for ultra-sensitive detection and enabling dynamic tumour burden monitoring and residual disease detection postoperatively [35].

Another crucial factor is the timing of ctDNA sampling, and is very much dependent on the outcome which is being investigated. ctDNA analyses can be applied for treatment response monitoring, as it acts as a real-time marker for minimal residual disease, and gives an indication of the treatment efficacy. The postoperative ctDNA status could aid in additional therapy decision-making after curative resection of CLRM, by identifying a group of patients with increased risk for recurrence. The main drawback of plasma sampling for the detection of ctDNA directly after surgery is the high concentration of non-cancerous cfDNA due to tissue damage from surgery [41]. This leads to the dilution of ctDNA, potentially affecting the sensitivity of detection, particularly in early postoperative samples. Although Reinert et al. showed that ctDNA could be detected as early as two days after surgery [42], there is now consensus that the timing of ctDNA sampling after surgery should ideally happen at least 2 weeks postoperatively [43]. Also, during surveillance, ctDNA has been shown to have potential in detecting recurrence ahead of clinical symptoms. Ogaard et al. found that 24% of patients had at least one inconclusive CT scan during surveillance, of whom 60% were eventually diagnosed with recurrence [17]. They conducted ctDNA analysis on plasma samples collected concomitantly with the inconclusive CT scans. The recurrence rate in patients with detectable ctDNA was significantly higher (positive predictive value 100%) than for the patients without detectable ctDNA (negative predictive value 75%).

Even though several questions remain, the clinical value of ctDNA has clearly been suggested in the colorectal cancer population. Large randomised trials failing to show an OS benefit of perioperative chemotherapy in resectable CRLM might be caused by the fact that not the entire CRLM population benefits, leading to the overtreatment of a select group of patients and a diluted effect in finding differences in the overall survival [44,45,46]. The potential of ctDNA may lie in risk stratification in terms of recurrence, which may contribute to a more personalised treatment strategy regarding perioperative chemotherapy. Stratification could potentially omit its usage and, therefore, the side effects of aggressive chemotherapy in CRLM patients at a low risk for recurrence. However, from the current available evidence on ctDNA in CRLM patients, conclusions cannot be drawn with regard to adjuvant treatment decision making for patients with undetectable ctDNA postoperatively. In stage II colon cancer, a ctDNA-guided treatment approach has been proven to reduce adjuvant chemotherapy without compromising the RFS [47,48]. A randomised trial investigating the role of adjuvant chemotherapy in both patients with and without detectable ctDNA after local treatment of CRLM has not been conducted and could contribute to a more personalised treatment of CRLM patients.

There are some limitations to this systematic review and meta-analysis, which should be considered. Various ctDNA detection methods were utilised in the included studies. In conducting the meta-analyses, all methods of ctDNA detection were allowed, given the lack of a current gold standard. Some studies investigated the tumour-informed approach through a specific gene mutational status (e.g., KRAS) and one study performed analyses with a patient-specific assay. Others performed tumour-agnostic screening using a predefined colorectal cancer panel. The impact of the method on the prognostic value of ctDNA is currently unknown and future research in this field is needed to reveal a gold standard. Secondly, the timing of blood sampling for ctDNA analysis also varied across the studies. A meta-analysis investigating the prognostic value of a pre-treatment sample (before neo-adjuvant therapy) could not be investigated due to lack of sampling at this time point. Most studies focused on a sample before and after surgery. Even within a sample time point, variation occurred, with some studies sampling right before resection and some within one month before resection. Similarly and more importantly, there was also heterogeneity in the sampling after surgery. Some studies collected blood samples within one week after surgery, which has been shown to be unreliable in the recent literature [43], and other studies collected samples within three months after surgery. These limitations highlight the importance of future research in ctDNA detection techniques and sample collection points.

## 5. Conclusions

This review has shown that patients with detectable ctDNA after surgery and after adjuvant chemotherapy have a higher risk of recurrence and a shorter overall survival compared to patients without ctDNA. From the available evidence, no definite association could be concluded between the preoperative ctDNA status and prognosis. The recurrence rate of patients with undetectable ctDNA after treatment was relatively high, indicating that at this point, adjuvant chemotherapy might not be omitted based on undetectable postoperative ctDNA alone. Improvements in ctDNA testing or novel biomarkers might omit the usage and therefore the side effects of aggressive chemotherapy in CRLM patients at a low risk for recurrence in the future, resulting in a higher quality of life.

## Figures and Tables

**Table 1:** Overall study characteristics and risk of bias (NSG: next generation sequencing, ddPCR: digital droplet polymerase chain reaction, nACT: neoadjuvant chemotherapy, ACT: adjuvant chemotherapy).

Author, Year	Study Design	Patients	Assay Type	NSG/PCR	Tumour-Informed/-Agnostic	Time Points (s)	Outcome	Risk of Bias
Newhook et al., 2022 [20] (USA)	Prospective single centre	48	Guardant variant classifier (23 genes)	NGS	Tumour-agnostic; for 38 patients tumour informed	Pre- and post-surgery	Long-term (oncologic) survival	Low
Nishioka et al., 2022 [28] (USA)	Retrospective single centre	105	NGS panel of 70 cancer-related genes	NGS	Tumour-agnostic	Post-surgery	Long-term (oncologic) survival	Moderate
Ogaard et al., 2022 [17] (Denmark)	Prospective single centre	96	Methylation profile	ddPCR	Tumour-agnostic	Pre-surgery, post-surgery, multiple in follow-up	Long-term (oncologic) survival	Moderate
Bolhuis et al., 2021 [25] (The Netherlands)	Prospective multicentre	23	KRAS and NRAS mutational status	ddPCR	Tumour-informed (specific gene mutation status)	Before nACT, pre-surgery and post-surgery	Long-term (oncologic) survival, adjuvant treatment response	Moderate
Kobayashi et al., 2021 [21] (Japan)	Prospective single centre	40	Guardant360^®^	NGS	Tumour-agnostic	Pre-surgery	Long-term (oncologic) survival	High
Tie et al., 2021 [27] (Australia)	Prospective multicentre	54	Patient personalised assay	NGS	Tumour-informed (patient specific)	Before nACT, pre-surgery and post-surgery (up to 2 years)	Long-term (oncologic) survival, adjuvant treatment response	Low
Wang et al., 2021 [24] (China)	Prospective single centre	91	ctDNA 451-gene NGS panel	NGS	Tumour-agnostic	Before nACT, pre-surgery, post-surgery and post-ACT (+ at disease progression)	Long-term (oncologic) survival, adjuvant treatment response	Low
He et al., 2020 [22] (China)	Prospective single centre	20	Coloncore panel	NGS	Tumour-agnostic	Pre- and post-surgery	Long-term (oncologic) survival	High
Polivka et al., 2020 [29] (Czech Republic)	Retrospective single centre	71	KRAS mutational status	ddPCR	Tumour-informed (specific gene mutation status)	Pre- and post-surgery	Long-term (oncologic) survival	High
Bidard et al., 2019 [26] (France)	Prospective multicentre	153	KRAS exon 2 mutational status	ddPCR	Tumour-informed (specific gene mutation status)	Before nACT, after 1 month of chemotherapy and pre-surgery	Long-term (oncologic) survival	Moderate
Narayan et al., 2019 [23] (USA.)	Propsective single centre	60	TP53 and APC mutational status	NGS and PCR	Tumour-informed (specific gene mutation status)	Intra-operative, 3–4 weeks post-surgery	Long-term (oncologic) survival, adjuvant treatment response	Moderate

## Data Availability

The original contributions presented in the study are included in the article and Supplementary Material. Further enquiries can be directed to the corresponding author.

## Supplementary Materials

The following supporting information can be downloaded at: https://www.mdpi.com/article/10.3390/cells12212520/s1. File S1: Search terms; Table S1: Quality Assessment QUIPS tool; Table S2: Reasons of bias QUIPS tool; Table S3: ctDNA measurement techniques; Figure S1: Sample timing, ctDNA detection rate and perioperative treatment per study; Figure S2: risk of publications bias summarized graphically through funnel plots.
