# Supplementary material for: Circulating Tumour DNA as Biomarker for Colorectal Liver Metastases: A Systematic Review and Meta-Analysis
Source: Cells. 2023 Oct 25;12(21):2520. doi: 10.3390/cells12212520 (PMC10647834; doi:10.3390/cells12212520)

Supplementary File S1 – Search terms

The following search was performed on the 26th of May 2023:

Embase

('colorectal liver metastasis'/de OR (('metastatic colorectal cancer'/exp OR 'colorectal cancer'/exp) AND ('liver metastasis'/de OR 'hepatectomy'/exp)) OR (((colorect* OR colo-rect* OR rect* OR colon*) NEAR/6 (liver* OR hepat*) NEAR/6 (metastas*)) OR ((((metatstat*) NEAR/6 (colorect* OR rect* OR colo*) NEAR/6 (canc* OR carcinom* OR tumor* OR tumour* OR neoplas* OR adenocarcinom* OR resect* OR unresect* OR excision*)) OR ((colorect* OR colo-rect* OR rect* OR colon*) NEAR/6 (metastas* OR cancer* OR tumour* OR tumor* OR neoplas* OR carcinom* OR adenocarcinom* OR resect* OR unresect* OR excision*))) AND (((liver OR hepat*) NEAR/6 metastas*) OR hepatect* OR ((liver* OR hepat*) NEAR/6 (resect* OR excis*))))):ab,ti,kw) **AND** ('circulating tumor DNA'/de OR 'DNA determination'/mj/de OR 'circulating free DNA'/de OR 'liquid biopsy'/de OR 'circulating tumor cell'/de OR (((free* OR circulat*) NEAR/3 (DNA*)) OR ctDNA* OR ct-DNA* OR cfDNA OR cf-DNA* OR ((liquid*) NEAR/3 (biops*)) OR ((circulat*) NEAR/3 (tumor* OR tumour* OR cancer* OR neoplas*)) OR CTC OR CTCs):ab,ti,kw) NOT ([Conference Abstract]/lim OR [Conference Review]/lim) AND [ENGLISH]/lim NOT ('case report'/de OR (case-report):ti) NOT ([animals]/lim NOT [humans]/lim)

Medline

(((exp Colorectal Neoplasms/) AND ((exp Liver Neoplasms/ AND exp Neoplasm Metastasis/) OR Hepatectomy/)) OR (((colorect* OR colo-rect* OR rect* OR colon*) ADJ6 (liver* OR hepat*) ADJ6 (metastas*)) OR ((((metatstat*) ADJ6 (colorect* OR rect* OR colo*) ADJ6 (canc* OR carcinom* OR tumor* OR tumour* OR neoplas* OR adenocarcinom* OR resect* OR unresect* OR excision*)) OR ((colorect* OR colo-rect* OR rect* OR colon*) ADJ6 (metastas* OR cancer* OR tumour* OR tumor* OR neoplas* OR carcinom* OR adenocarcinom* OR resect* OR unresect* OR excision*))) AND (((liver OR hepat*) ADJ6 metastas*) OR hepatect* OR ((liver* OR hepat*) ADJ6 (resect* OR excis*))))).ab,ti,kf.) **AND** (Circulating Tumor DNA/ OR *Sequence Analysis, DNA/ OR Cell-Free Nucleic Acids/ OR Liquid Biopsy/ OR Neoplastic Cells, Circulating/ OR (((free* OR circulat*) ADJ3 (DNA*)) OR ctDNA* OR ct-DNA* OR cfDNA OR cf-DNA* OR ((liquid*) ADJ3 (biops*)) OR ((circulat*) ADJ3 (tumor* OR tumour* OR cancer* OR neoplas*)) OR CTC OR CTCs).ab,ti,kf.) NOT (news OR congres* OR abstract* OR book* OR chapter* OR dissertation abstract*).pt. AND (english).lg NOT (Case Reports/ OR (case-report).ti.) NOT (exp animals/ NOT humans/)

Cochrane (Ovid=same)

((((colorect* OR colo NEXT rect* OR rect* OR colon*) NEAR/6 (liver* OR hepat*) NEAR/6 (metastas*)) OR ((((metatstat*) NEAR/6 (colorect* OR rect* OR colo*) NEAR/6 (canc* OR carcinom* OR tumor* OR tumour* OR neoplas* OR adenocarcinom* OR resect* OR unresect* OR excision*)) OR ((colorect* OR colo NEXT rect* OR rect* OR colon*) NEAR/6 (metastas* OR cancer* OR tumour* OR tumor* OR neoplas* OR carcinom* OR adenocarcinom* OR resect* OR unresect* OR excision*))) AND (((liver OR

hepat*) NEAR/6 metastas*) OR hepatect* OR ((liver* OR hepat*) NEAR/6 (resect* OR excis*))))):ab,ti) **AND** ((((free* OR circulat*) NEAR/3 (DNA*)) OR ctDNA* OR ct NEXT DNA* OR cfDNA OR cf NEXT DNA* OR ((liquid*) NEAR/3 (biops*)) OR ((circulat*) NEAR/3 (tumor* OR tumour* OR cancer* OR neoplas*)) OR CTC OR CTCs):ab,ti) NOT "conference abstract":pt

Web of Science

TS=(((((colorect* OR colo-rect* OR rect* OR colon*) NEAR/5 (liver* OR hepat*) NEAR/5 (metastas*)) OR ((((metatstat*) NEAR/5 (colorect* OR rect* OR colo*) NEAR/5 (canc* OR carcinom* OR tumor* OR tumour* OR neoplas* OR adenocarcinom* OR resect* OR unresect* OR excision*)) OR ((colorect* OR colo-rect* OR rect* OR colon*) NEAR/5 (metastas* OR cancer* OR tumour* OR tumor* OR neoplas* OR carcinom* OR adenocarcinom* OR resect* OR unresect* OR excision*))) AND (((liver OR hepat*) NEAR/5 metastas*) OR hepatect* OR ((liver* OR hepat*) NEAR/5 (resect* OR excis*)))))) **AND** ((((free* OR circulat*) NEAR/2 (DNA*)) OR ctDNA* OR ct-DNA* OR cfDNA OR cf-DNA* OR ((liquid*) NEAR/2 (biops*)) OR ((circulat*) NEAR/2 (tumor* OR tumour* OR cancer* OR neoplas*)) OR CTC OR CTCs)) NOT ((animal* OR rat OR rats OR mouse OR mice OR murine OR dog OR dogs OR canine OR cat OR cats OR feline OR rabbit OR cow OR cows OR bovine OR rodent* OR sheep OR ovine OR pig OR swine OR porcine OR veterinar* OR chick* OR zebrafish* OR baboon* OR nonhuman* OR primate* OR cattle* OR goose OR geese OR duck OR macaque* OR avian* OR bird* OR fish*) NOT (human* OR patient* OR women OR woman OR men OR man))) AND LA=(English) NOT DT=(Meeting Abstract OR Meeting Summary) NOT TI=(case-report)

Google Scholar

"colorectal|rectal|colon liver|hepatic metastasis|metastases" "circulating|ct|cf DNA"|"cell free DNA"|ctDNA|cfDNA|"circulating tumor|tumour DNA|cell"|"cell free tumor|tumour DNA"|"liquid biopsy"

'colorectal|rectal|colon liver|hepatic metastasis|metastases' 'circulating|ct|cf DNA'|'cell free DNA'|ctDNA|cfDNA|'circulating tumor|tumour DNA|cell'|'cell free tumor|tumour DNA'|'liquid biopsy'

Table *<u>S1 – Quality Assessment QUIPS tool</u>*

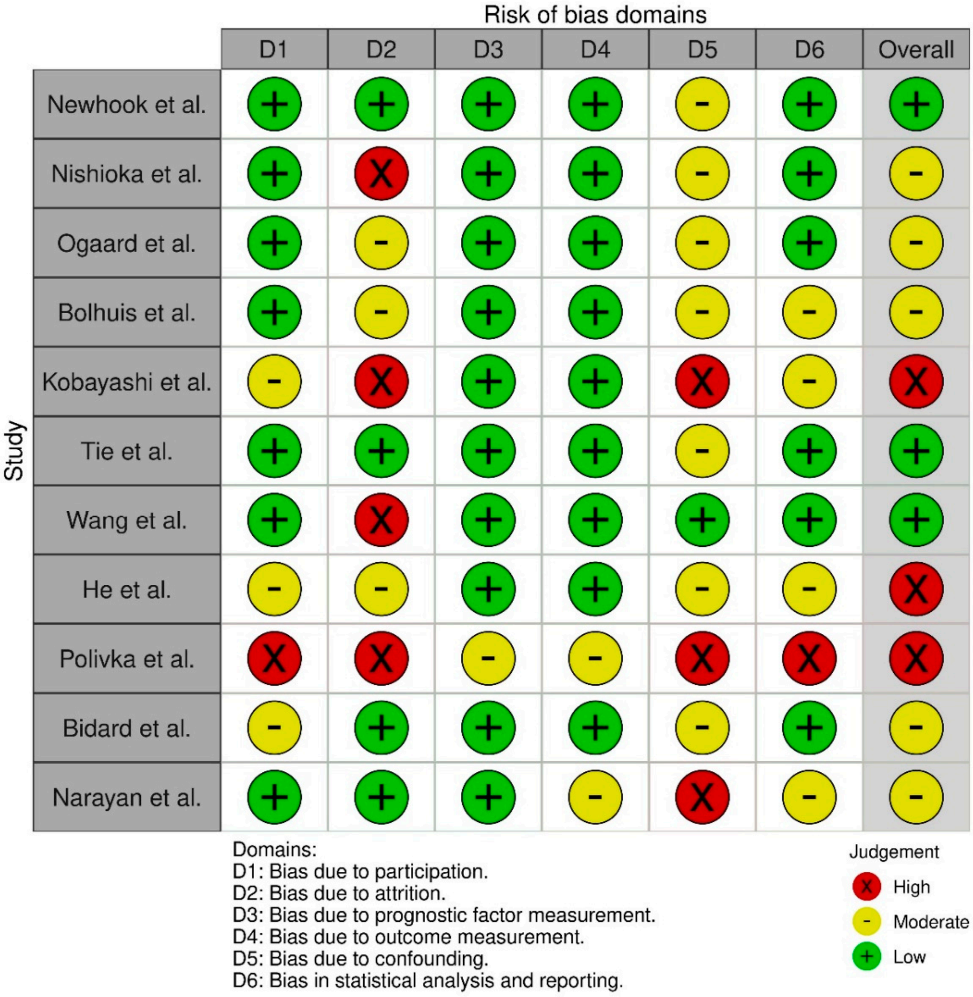

Table *<u>S2 – Reasons of bias QUIPS tool</u>*

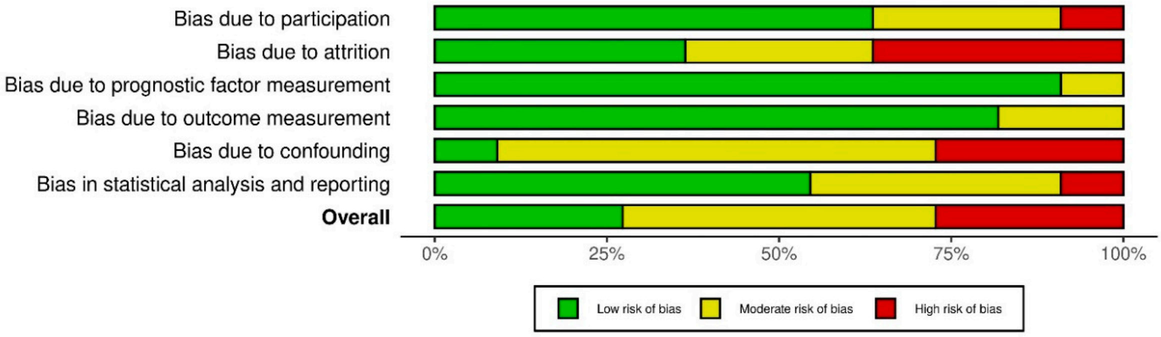

Table *<u>S3 – ctDNA measurement techniques (next page)</u>*

Author, year	NGS/PCR	Target / assay	Tumour-informed/agnostic	Tube type	Plasma isolation	cfDNA isolation	plasma volume	interval btw surgery and postop sample	Risk of Bias
Newhook et al. 2022 [20] (U.S.A.)	NGS	Guardant variant classifier (23 genes)	Tumour-agnostic; for 38 pt Tumour-informed	EDTA	dual centrifugation directly following blood draw	unknown	5ml blood > ~2 ml plasma	18 days (IQR 13-68)	Low
Nishioka et al. 2022 [28] (U.S.A.)	NGS	NGS panel of 70 cancer-related genes + part with Guardant assay	Tumour-agnostic	unknown	unknown	unknown	unknown	Within 180 days	Moderate
Ogaard et al. 2022 [17] (Denmark)	ddPCR	Methylation profile	Tumour-agnostic	K2-EDTA	10 min at 3,000g within 2 hrs, plasma 10 min at 3,000g, room temp	qiaAMP	8 mL	Within 90 days	Moderate
Bolhuis et al. 2021 [25] (The Netherlands)	ddPCR	KRAS and NRAS mutational status	Tumour-informed (specific gene mutation status)	cell-stabilising BCT tube	1,700 g for 10 min, 20,000 g 10 min	QIAsymphony	10 ml blood > ~4 ml plasma	After 3 months (maximally 100 days)	Moderate
Kobayashi et al. 2021 [21] (Japan)	NGS	Guardant360®	Tumour-agnostic	unknown	unknown	qiaAMP	2 ml	No post-surgery sample	High
Tie et al. 2021 [27] (Australia)	NGS	Patient personalised assay	Tumour-informed (patient specific)	EDTA	centrifuged twice at 1,200g and 1,800g; speed unknown	qiaAMP	10 ml	4-10 weeks	Low
Wang et al. 2021 [24] (China)	NGS	ctDNA 451-gene NGS panel	Tumour-agnostic	K2-EDTA	1,600 g for 10 min, 10,000 g for 10 min; speed unknown	qiaAMP	3-3.5 ml	Median time of 38 days after surgery	Low
He et al. 2020 [22] (China)	NGS	Coloncore panel	Tumour-agnostic	STRECK	2400 g for 10 min and plasma at 16000xg for 5 min	qiaAMP	10ml blood > ~4ml plasma	Within 7 days	High
Polivka et al. 2020 [29] (Czech Republic)	ddPCR	KRAS mutational status	Tumour-informed (specific gene mutation status)	K3-EDTA	950 rcf at 10 min at 4 °C and then at 11,000 rcf at 10 min at 4 °C	qiaAMP	1 ml	1 day	High
Bidard et al. 2019 [26] (France)	ddPCR	KRAS exon 2 mutational status	Tumour-informed (specific gene mutation status)	CellSave®	10 min at 820 g within 3 hrs, 16,000 g for 10 min at 15 °C	qiaAMP	4 ml	No post-surgery sample	Moderate
Narayan et al. 2019 [23] (U.S.A.)	NGS and PCR	TP53 and APC mutational status	Tumour-informed (specific gene mutation status)	Cell-Free DNA BCT tube (STRECK)	according to reference only 1 centrifugation step	according to reference qiaAMP	unknown	3-4 weeks	Moderate

ctDNA: circulating tumour DNA, NSG: next generation sequencing, ddPCR: digital droplet PCR, PCR: polymerase chain reaction

Figure *S1 – Sample timing, ctDNA detection rate and perioperative treatment per study.* *Blood sample tubes were used to illustrate when sampling took place. Additionally, when ctDNA positivity rates were provided by the authors, these were included in yellow. Neoadjuvant and adjuvant treatment is illustrated by the green bars, including the amount of patients receiving the treatment.*

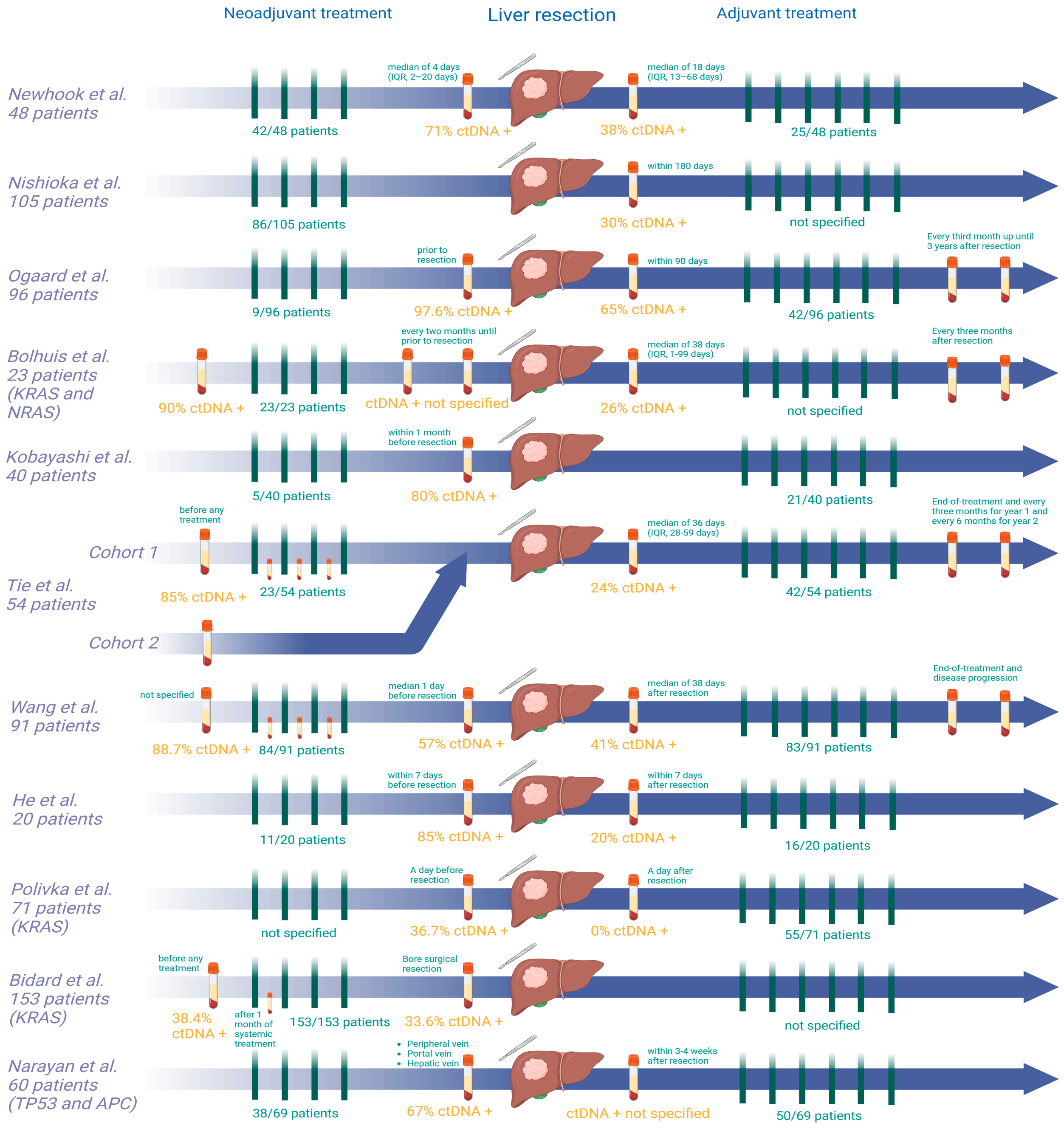

Figure *S2 – Funnel plots. (Abbreviations: RFS, Recurrence-free Survival; OS, Overall Survival; SE, Standard Error)*

Preoperative sample, RFS

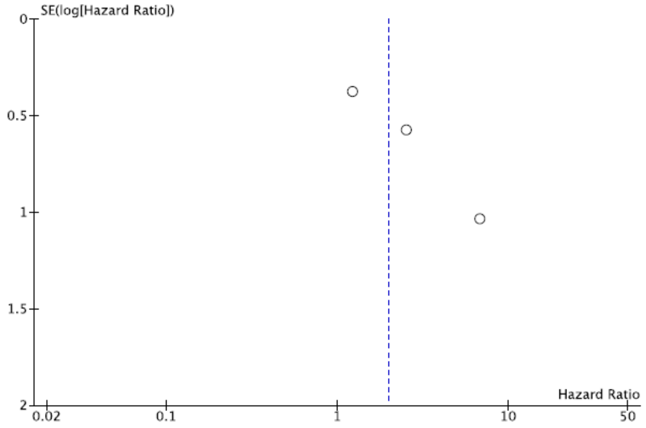

Postoperative sample, RFS

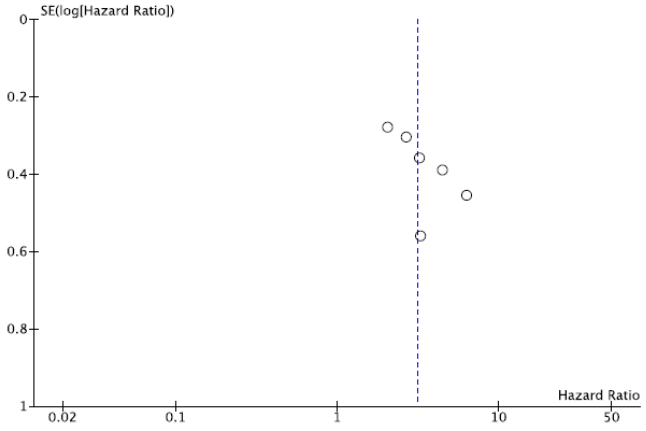

Preoperative sample, OS

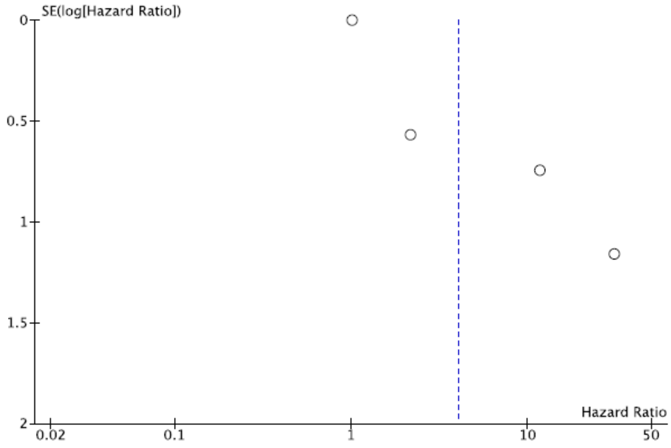

Postoperative sample, OS

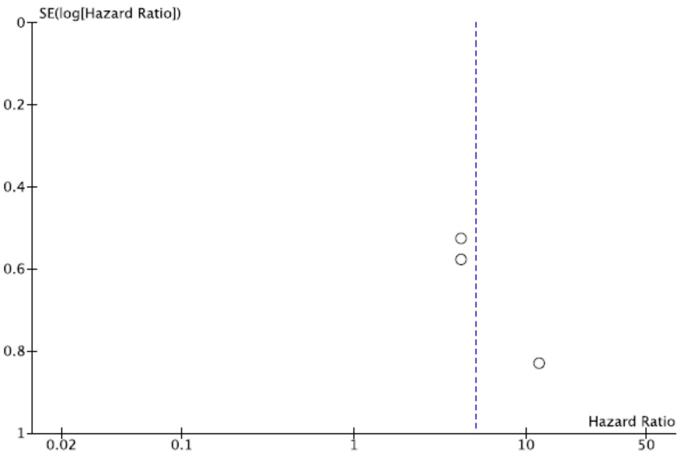

End of adjuvant therapy sample, RFS

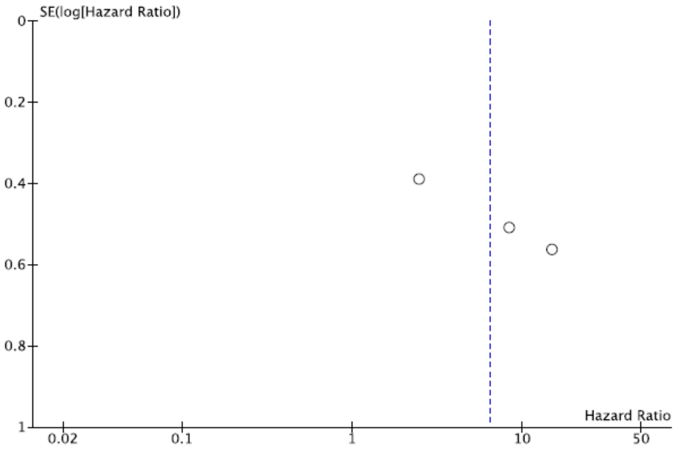

Supplement: Supplementary file 1 [file cells-12-02520-s001.zip › cells-2631056-supplementary.pdf]
